# Editorial: Next generation chemical risk assessment: integration of advances in toxicology, biology and computation

**DOI:** 10.3389/ftox.2024.1440229

**Published:** 2024-07-02

**Authors:** Kan Shao, Chao Ji, Bernard Gadagbui

**Affiliations:** ^1^ Indiana University, Bloomington, IN, United States; ^2^ Indiana University Bloomington, Bloomington, IN, United States; ^3^ Agency for Toxic Substances and Disease Registry, Atlanta, GA, United States; ^4^ Toxicology Excellence for Risk Assessment (TERA), Cincinatti, OH, United States

**Keywords:** next generation risk assessment (NGRA), computational toxicology, high-throughput (HT) screening, deep learning, endocrine disruptors

Recent technological advancements in toxicology, such as high-throughput screening assays, -omics technology, and machine learning based computational algorithms, have created unique and promising opportunities to fundamentally improve risk assessment through more rigorous hazard characterization, more effective dose-response assessment, and more accurate exposure assessment. Given the situation that the large number of chemicals created and introduced into the environment has substantially exceeded the capacity of current risk assessment framework, a next generation risk assessment that aims to efficiently and cost effectively evaluate chemical safety has been proposed and is being developed by a multi-sector risk assessment community. The main objective of the next generation risk assessment will be achieved primarily by incorporating new chemical testing data and advanced molecular and systems biology technologies to accelerate and modernize or replace the traditional animal-based risk assessment.

However, the next generation risk assessment faces substantial scientific challenges and uncertainties, including the development of reliable molecular predictive indicators of effects for a large variety of chemicals, understanding the adverse outcome pathways needed to characterize toxicological mechanisms, and quantifying the uncertainty and variability embedded in the new data types and modeling methodologies. The Research Topic with the theme “Next-Generation Chemical Risk Assessment” covers several important topics in the field, including database and computational algorithms to support more advanced modeling strategies, and high-throughput assay to assist in understanding chemical-organism interaction. All contributions to this Research Topic were responses to a public call for submissions issued by the editors through Frontiers in Toxicology. All submitted papers were initially reviewed by the editors to determine their suitability for publication in this Research Topic. Then, each paper underwent the peer review process managed by the journal.

The Research Topic consists of three original research papers and one review paper. The first paper (Feshuk et al.) introduces the Toxicity Reference Database (ToxRefDB) v2.1 which updates its previous version by correcting the compilation error that might result in inadvertently omitted effects from the database. The improved functionalities will certainly enhance the utility of ToxRefDB as an important resource for classical *in vivo* toxicological information. Currently, ToxRefDB has *in vivo* study data covering more than 1,100 chemicals collected from over 5,900 studies. To ensure the consistency of the datasets in ToxRefDB, the database contains information about chemical name, study designs (such as dosing, duration, exposure route), animal information, as well as treatment-related effects (which are controlled and standardized by effect vocabulary). ToxRefDB can provide critical information to justify the scientific plausibility of *in vitro* high-throughput screening of chemicals and serve as a reference to support retrospective and predictive toxicology applications.

The second paper (Mostafa and Chen) reviews the application of deep learning (DL) in quantitative structure-activity relationship (QSAR) modeling to predict drug-induced liver injury (DILI). The authors comprehensively evaluated a variety of DL algorithms regarding their scalability, generalizability, and interpretability and compared the performance of the DL algorithms with traditional machine learning approaches. The study found that the DL algorithms had important potential to improve DILI predictions and consequently create foundations for DILI risk mitigation by developing more accurate predictive models. However, it is also important to note that the advantages of DL depend on the specifics of the datasets and problems. The significance of the review is that the advantages and limitations of DL methodologies in QSAR modeling for DILI toxicity prediction were objectively evaluated, which is an important initial step to better understand how DL methods can be more effectively applied in predictive toxicology and risk assessment.

Paper 3 (Joe Bever et al.) and Paper 4 (Nelms et al.) came from the same research group to discuss two applications of high-throughput assays within the U. S. Environmental Protection Agency’s (EPA’s) Endocrine Disruptor Screening Program (EDSP). Paper three evaluated a few subsets of assays in the androgen receptor (AR) pathway model to determine the smallest subset of assay batteries that can still maintain sensitivity across a broad chemical space. Such an optimized data processing method will not only minimize the cost of assays but also provide a uniform approach to assess the performance of reduced assay batteries from the original AR pathway model. On the other hand, Paper four utilized the k-nearest neighbors-based chemical clustering approach to investigate the feasibility of a reduced estrogen receptor (ER) model for screening endocrine disrupting chemicals. The results suggested that the existing 4-assay model can perform consistently well for a variety of chemicals with diverse structures. These two studies illustrated how improved modeling approaches may optimize the use of high-throughput assays for more efficient screening of endocrine disrupting chemicals, which will have substantial utility in the next generation risk assessment.

